# Epidemiological, clinical, and microbiological characteristics of listeriosis in Qatar: A retrospective study

**DOI:** 10.5339/qmj.2025.87

**Published:** 2025-09-18

**Authors:** Sreethish Sasi, Wael Goravey, Sara Al Balushi, Emad Ibrahim, Javed Iqbal, Abdellatif Al Khal, Muna Al Maslamani, Gawahir A. Ali

**Affiliations:** 1 Infectious Diseases Division, Department of Medicine, Communicable Diseases Center, Hamad Medical Corporation, Doha, Qatar; 2 Department of Epidemiology, Communicable Diseases Center, Hamad Medical Corporation, Doha, Qatar; 3 Microbiology Division, Department of Laboratory Medicine and Pathology, Hamad Medical Corporation, Doha, Qatar; 4 Biomedical Research Center, Qatar University, Doha, Qatar; 5 Department of Nursing, Communicable Diseases Center, Hamad Medical Corporation, Doha, Qatar; 6 Weil Cornell Medical College, Doha, Qatar; 7 College of Medicine, Qatar University, Doha, Qatar *Email: ssasi7@hamad.qa

**Keywords:** Listeriosis, epidemiology, pregnancy, bacteremia, antimicrobials, State of Qatar

## Abstract

**Introduction:**

*Listeria monocytogenes* is a bacterium found in raw foods and water and causes severe infections in immunocompromised, pregnant women, and the elderly. Although the incidence of listeriosis is low, it is a life-threatening disease with a case–fatality rate of 20% to 30% and numerous complications, including central nervous system (CNS) infections and maternal-fetal transmission. The purpose of this study was to determine the epidemiology, clinical features, and outcomes of listeriosis in Qatar over 10 years.

**Methods:**

This retrospective cohort study was conducted at Hamad Medical Corporation, the main public healthcare provider in Qatar, analyzing laboratory-confirmed *L*. *monocytogenes* bloodstream infections from May 1, 2011, to November 26, 2021. The data were collected from the electronic medical records system and included demographic data, clinical features, microbiology, management, and outcome. The study was approved by the Institutional Review Board (MRC-01-21-1023). The inclusion criteria for the study were positive blood cultures and clinical signs of infection. Descriptive statistics and comparative analyses were used in the statistical analyses.

**Results:**

The study involved 35 cases with a median age of 39 years and 77.14% female. Pregnancy-associated cases were 22.86% with high fetal morbidity, including 33.33% stillbirth and 55.56% preterm delivery. Twenty percent of the patients had clinical features of meningoencephalitis. Although the source of infection could not be determined, it is known that *Listeria monocytogenes* is most commonly transmitted through contaminated food products. All the isolates were sensitive to ampicillin, co-trimoxazole, and meropenem. The 30- and 90-day mortality rates were 2.86% and 14.26%, respectively, and age (60 years and above) and meningoencephalitis were independent predictors of mortality.

**Conclusion:**

This study offers significant information about listeriosis in Qatar, characterized by a higher incidence of pregnancy-associated cases and a lower incidence of CNS involvement than in other countries. These findings also show the gaps in antimicrobial resistance surveillance and the foodborne transmission in the Middle East. Even though all mothers recovered fully, the adverse fetal outcomes stress the importance of preventive measures and enhanced food safety measures. Future research should focus on molecular characterization, source attribution, and antimicrobial resistance monitoring to enhance infection control and public health interventions, ultimately mitigating the impact of listeriosis in the region.

## 1. INTRODUCTION

*Listeria (L.) monocytogenes* is a gram-positive facultative intracellular bacterium^1^ and a pathogen of importance in the food industry that causes severe infections in immunocompromised individuals, pregnant women, neonates, and the elderly.^2^ Although the incidence is lower (0.1 to 1.1 cases per 100,000 population) than that of other bacterial pathogens,^2–4^ *L*. *monocytogenes* is characterized by a high case-fatality rate of 20% to 30%^5^ and the ability to cause severe central nervous system (CNS) infections, sepsis, and maternal-fetal complications.^2,5^ The pathogen is adaptive to cold storage conditions and can escape from the host immune system by remaining intracellular, which makes it a significant public health issue.^6^ Despite the fact that listeriosis has been most well described in high-income countries^7^ because of effective food safety interventions, the epidemiological data are incomplete for many areas, including the Middle East. Qatar, one of these countries, with a large expatriate population,^8^ has no specific data on the incidence and nature of listeriosis, although it is a notifiable disease. This lack of regional studies hampers public health efforts to reduce the impact of *L*. *monocytogenes*.

The pathogenesis of *L*. *monocytogenes* is known to involve several virulence factors that assist in the adherence of target cells, intracellular survival, and spread.^9^ The bacterium invades epithelial cells via surface proteins such as Internalins (InlA and InlB) that interact with E-cadherin to allow the cell entry.^9,10^ After being taken up by a cell, *L*. *monocytogenes* escapes from the phagosome by means of listeriolysin O, a protein toxin that perforates vacuolar membranes and permits bacterial growth in the cytosol. The pathogen subverts the host’s actin polymerization machinery to propel itself from cell to cell, thus ensuring its spread while remaining undetected by the immune system.^9,10^ Its capacity to cross the blood-brain barrier and the placenta leads to severe clinical complications such as meningitis, encephalitis, sepsis, and maternofetal infections.^5–7,11^ Listeriosis presents in various clinical forms depending on host susceptibility.^7^ It can cause febrile gastroenteritis that is usually mild and self-limiting in healthy individuals, but in neonates, the elderly, and immunosuppressed, it can progress to septicemia, meningitis, or meningoencephalitis with high mortality.^5,12^ Listeriosis in pregnancy is of particular concern because of the sequelae of fetal death, preterm labor, and neonatal sepsis, with transmission occurring either hematogenously or during birth.^6^

The incidence of listeriosis varies worldwide and is between 0.1 and 1.1 cases per 100,000 population^7^ and is higher in pregnant women and human immunodeficiency virus–infected individuals.^5,7^ Large foodborne outbreaks, which are usually associated with contaminated dairy products and ready-to-eat meats as well as fresh produce, highlight the importance of continuing surveillance.^13^ Very few studies have been conducted on the burden of listeriosis in the Middle East and Qatar, with its unique population and dietary patterns presenting additional difficulties. The frequency of *L*. *monocytogenes* in dairy products across the region is estimated at 3.5%, with the highest frequency reported in Jordan (17.6%) and the lowest in Iraq (1.6%).^14^ Raw milk is especially vulnerable; contamination has been observed to be as high as 5.8%.^14^ According to the reports from North African countries, *L*. *monocytogenes* is more frequent in animal-sourced foods than in plant-sourced foods.^15^ Lack of data on serotypes and strain diversity of *L*. *monocytogenes* in the region and the need for improved surveillance have been identified.^16^

Hamad Medical Corporation (HMC), the main public healthcare provider in the State of Qatar, comprises 14 hospitals, including general and specialty care institutions, with over 2,500 beds. It delivers secondary and tertiary healthcare services to nearly 3 million residents.^17^ This retrospective cohort study examines *L*. *monocytogenes* bloodstream infections at HMC from 2011 to 2021, to analyze epidemiological, microbiological, and clinical features, identify risk factors for poor outcomes, guide interventions, and enhance prevention, diagnosis, and treatment strategies, particularly for vulnerable groups.

## 2. MATERIALS AND METHODS

This retrospective cohort study analyzed blood culture-confirmed cases of *L*. *monocytogenes* infection at HMC in Qatar between May 1, 2011, and November 26, 2021. HMC, the largest tertiary healthcare provider in Qatar, serves a diverse population, including expatriate workers and residents.^1^ Data were extracted from the electronic medical records system and laboratory databases.

- **Inclusion criteria:**
  1. At least one positive culture for *L*. *monocytogenes* from blood.
  2. Clinical evidence of infection compatible with listeriosis, such as sepsis, meningitis, encephalitis, or pregnancy-associated listeriosis.
- **Exclusion criteria:**
  1. Duplicate cases from repeated positive blood cultures in the same patient during a single clinical episode.
  2. Cases without clinical signs consistent with listeriosis (e.g., incidental colonization or contamination without symptoms).
  3. Patients whose culture-positive specimens were not from blood.

All eligible cases during the study period were included without sample size estimation. Data collection utilized a structured case report form, capturing demographic details (age, sex, nationality), and comorbidities (diabetes, malignancy, chronic kidney disease, immunosuppression, and pregnancy status), risk factors (use of proton pump inhibitors (PPIs), recent antibiotic exposure, and food consumption history if documented), and clinical presentations (fever, neurological symptoms, sepsis, meningitis, or pregnancy-related complications). Laboratory and microbiological data included white blood cell (WBC) count, C-reactive protein (CRP), renal and liver function tests at presentation, and microbiological identification using VITEK-2® (bioMérieux, Marcy-l’Étoile, France) or Matrix Assisted Laser Desorption Ionization - Time of Flight (MALDI-TOF). Antimicrobial susceptibility testing was performed via disk diffusion and broth microdilution, following Clinical and Laboratory Standards Institute (CLSI) guidelines, with resistance patterns assessed for penicillins, aminoglycosides, cotrimoxazole, and meropenem. Treatment and outcome data encompassed empirical and definitive antibiotic therapy (e.g., ampicillin ± gentamicin, cotrimoxazole, meropenem), time to bacteremia clearance based on serial cultures, length of hospital stay (LOS), need for intensive care unit (ICU) admission, and 30-day and 90-day mortality rates. This study was conducted in accordance with the principles of the Declaration of Helsinki and was approved by the Institutional Review Board under the Medical Research Center (MRC) of HMC, Qatar, under approval number MRC-01-21-1023.

### 2.1 Statistical Analysis

All data were entered into Microsoft Excel and analyzed using SPSS Statistics version 26 (IBM Corp., Released 2019. IBM SPSS Statistics for Windows, Version 26.0; IBM Corp., Armonk, NY). Descriptive statistics were used to summarize the data, with continuous variables such as age, WBC count, and hospital stay reported as medians with interquartile ranges (IQR), while categorical variables like mortality, comorbidities, and pregnancy status were presented as frequencies and percentages. Comparative analyses were conducted to assess differences between survivors and non-survivors, employing the Mann-Whitney U test for continuous variables and the Chi-square or Fisher’s exact test for categorical variables. To identify independent predictors of mortality, a multivariate logistic regression model was used, incorporating variables with a p-value < 0.05 from univariate analysis. Survival analysis was performed using Kaplan-Meier survival curves to assess time to mortality, stratified by ICU admission and immunocompromised status, with the log-rank test used to compare survival distributions between groups. A *p*-value < 0.05 was considered statistically significant for all analyses.

## 3. RESULTS

**Figure 1:**
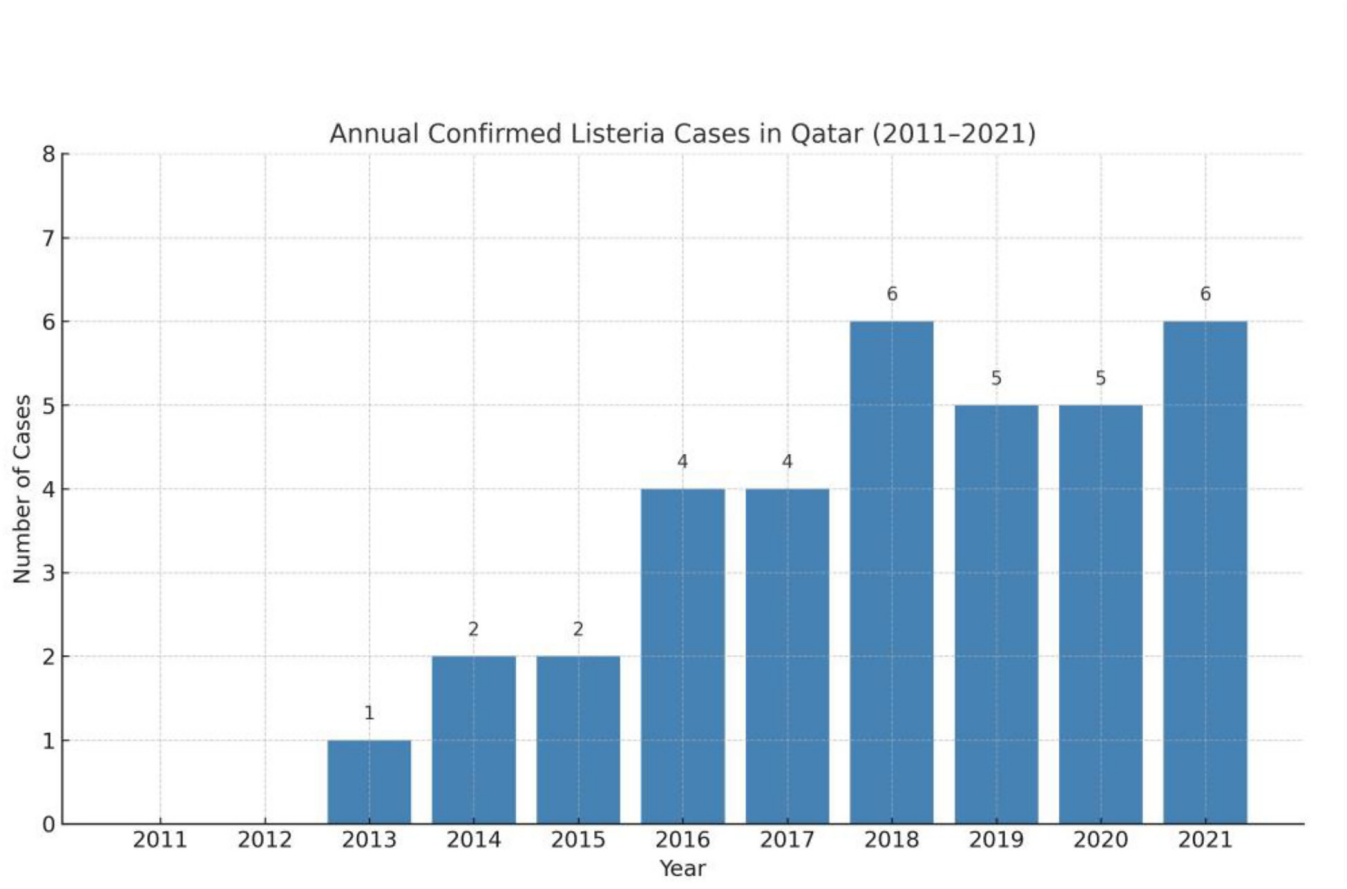
Number of confirmed *Listeria monocytogenes* cases per year from 2011 to 2021 at Hamad Medical Corporation, Qatar.

A total of 35 cases of laboratory-confirmed *L*. *monocytogenes* bloodstream infections were identified at HMC in Qatar between May 1, 2011, and November 26, 2021. Year-wise distribution of the number of cases is shown in Figure 1. The median age of patients was 39 (IQR: 28–62) years, with 77.14% (*n* = 27) being female. Most patients (65.71%, *n* = 23) were from the Eastern Mediterranean Region, while others were from South or Southeast Asia. Comorbidities were common, with 40% (*n* = 14) having diabetes mellitus, 28.57% (*n* = 10) having chronic kidney disease (CKD), and 25.71% (*n* = 9) having malignancy. Pregnancy was noted in 22.86% (*n* = 8) of cases, and 37.14% (*n* = 13) had recent PPI use, which may have increased susceptibility to infection. Twenty percent (*n* = 7) of the patients had clinical features of meningoencephalitis. Fever (91.43%, *n* = 32) was the most common symptom, while neurological symptoms such as altered mental status and seizures were observed in 17.14% (*n* = 6) of cases (Table 1). Among pregnancy-associated cases (n = 8), fetal morbidity was high (87.50%, *n* = 7), with 33.33% (*n* = 2) resulting in stillbirth and 55.56% (*n* = 5) involving preterm delivery. A unique case of twin pregnancy resulted in one fetal loss while the other fetus survived. All mothers recovered following appropriate antimicrobial therapy (Tables 2 and 3).

The median WBC count was 12,800 cells/mm³ (IQR: 9,200–15,300), with CRP more than 30 in 85.71% (*n* = 30) of cases. Renal impairment was noted in 22.86% (*n* = 8) at diagnosis. All *L*. *monocytogenes* isolates were susceptible to ampicillin, cotrimoxazole, and meropenem, as per CLSI guidelines (Table 2). Treatment involved intravenous antibiotics, with ampicillin (85.71%, *n* = 30) being the most used agent, often combined with gentamicin (57.14%, *n* = 20). Cotrimoxazole was administered in 11.43% (*n* = 4) of cases, primarily as step-down therapy. Bacteremia cleared within 72 hours in 80.00% (*n* = 28) of patients, and the median hospital LOS was 4 (IQR: 2–7) days. Mortality rates were 2.86% (*n* = 1) at 30 days and 14.26% (*n* = 5) at 90 days, with ICU admission required in 11.43% (*n* = 4) of cases, predominantly for meningoencephalitis (Table 2). Pregnancy-associated cases had a 100% maternal survival rate, though fetal complications (88.89%) were significant (eight out of nine fetuses, including a twin pregnancy; Table 3).

Comparative analysis between survivors (*n* = 30) and non-survivors (*n* = 5), after 1 year of infection (Table 4), revealed that non-survivors were older (median age: 63 vs. 36 years, *p* = 0.003), more likely to have CKD (80.00% vs. 20.00%, *p* = 0.002), and more frequently presented with meningoencephalitis (60.00% vs. 13.33%, *p* = 0.005) or required ICU admission (80.00% vs. 3.33%, *p* < 0.001). Multivariate logistic regression identified age ≥ 60 years (odds ratio [OR], 5.8 [95% CI, 2.1–14.7]; *p* = 0.001) and meningoencephalitis (OR, 4.2 [95% CI, 1.8–9.9]; *p* = 0.003) as independent predictors of mortality. Kaplan-Meier survival curves demonstrated significantly worse outcomes for patients with meningoencephalitis (log-rank test: *p* < 0.01) and those who were immunocompromised (log-rank test: *p* = 0.02).

## 4. DISCUSSION

This retrospective study provides a comprehensive epidemiological and clinical analysis of listeriosis in Qatar, examining 35 cases over 10 years. The annual incidence of listeriosis in Qatar was 0.1 to 0.2 cases per 100,000 population, compared to a global incidence of 0.1 to 1.1 cases per 100,000 population.^2^ Our findings highlight key demographic groups, risk factors, clinical manifestations, treatment outcomes, and mortality predictors, offering insights into regional trends while comparing with global and Middle Eastern data.

### 4.1 Demographics and Risk Factors

In our study, the median age of the patients was 39 years, which is lower than that reported in Western countries, where listeriosis is largely seen among the elderly over 60 years of age.^7^ In our cohort, 77.14% of cases were in females, with pregnancy-associated infections accounting for 22.86%, which highlights the importance of materno-fetal transmission.^6^ This percentage is consistent with that reported from Europe (17%–30%) but is higher than that of some Asian studies.^6^ The presence of comorbidities such as diabetes mellitus (40%), chronic kidney disease (28.57%), and malignancy (25.71%) was similar to that noted in other studies conducted globally, and these are known to be risk factors for listeriosis.^2,3,12^ Further, 37.14% of patients had recent PPI use, a known risk factor for listeriosis.^1,2,5,7^

### 4.2 Clinical Presentation and Outcomes

The incidence of CNS infections in our study (20%) was lower than the global reports (24%–47%), probably due to regional variations in immune response and early interventions.^4,11^ Compared to a Chinese study of 59 cases where 42.37% had neuroinvasive infections,^12^ CNS involvement in Qatar was lower (20%), which might be related to the differences in healthcare systems and diagnostic methods. In pregnancy-associated cases, maternal survival was 100% which is attributable to effective antimicrobial treatment. However, fetal complications and mortality were still high, which suggests that there is a need for targeted maternal screening and preventive measures.

### 4.3 Pregnancy-Related Listeriosis

In our study, pregnancy-related listeriosis had high fetal complications, including stillbirth (33.33%) and preterm delivery (55.56%). These findings are consistent with a study from Slovakia, where pregnancy-associated listeriosis accounted for a large percentage of the cases with poor neonatal outcomes.^6^ A unique case of twin pregnancy with one fetal death also demonstrates the destructive impact of *L*. *monocytogenes* on fetal health. Globally, early-onset neonatal listeriosis has a high case-fatality rate (20%–30%).^19^ Preterm infants are particularly at high risk of death, while late-onset disease may have long-term neurological complications.^6,20^ Our study confirms these findings and stresses the necessity of making the right diagnosis at an early stage to prevent fetal morbidity and mortality.

### 4.4 Microbiological Findings and Antibiotic Resistance

Our findings show that all the isolates were fully susceptible to ampicillin, cotrimoxazole, and meropenem, but gentamicin susceptibility was 14.29%. MICs were interpreted following the CLSI guidelines. This is consistent with the global findings that penicillin, ampicillin, and meropenem are currently the most effective therapies.^2^ But resistance to ampicillin and penicillin was observed in up to 15.22% of the isolates from Inner Eurasia.^21^ The surveillance of antimicrobial resistance in *L*. *monocytogenes* is scarce in the Middle East, though studies from Iran show low levels of resistance in dairy-related isolates.^14–16^ Because of the high incidence of dairy-associated Listeria cases, further molecular characterization of the regional strains is important.^14^

### 4.5 Mortality and Predictors of Poor Outcomes

The 90-day mortality rate was 14.26%, consistent with global estimates of 10% to 30%.^2–5, 21^ Advanced age (≥60 years) and meningoencephalitis were independent predictors of mortality, which aligns with findings from Europe and Asia.^4–6^ Non-survivors were more likely to require ICU admission (80%) and had significant comorbidities. Compared to the mortality rate in a Chinese cohort (8.47%),^12^ our study observed a higher mortality rate (14.26%), which may reflect differences in healthcare infrastructure, case severity, or underlying comorbidities. Interestingly, although the Chinese cohort had a high burden of comorbidities (90.7%), including malignancy and immunosuppression, the lower mortality observed may also reflect differences in diagnostic timing, case mix, or therapeutic interventions.

### 4.6 Comparison with Middle Eastern Data

Reports from the Middle East indicate that *L*. *monocytogenes* is frequently isolated from animal-sourced foods but is less commonly reported in plant-based foods.^7^ In North Africa, *L*. *monocytogenes* is well-documented in dairy products, but serotype distribution and genetic diversity remain poorly characterized in the Arab region.^8^ The prevalence of *L*. *monocytogenes* in dairy products in the Middle East is estimated at 3.5%, with Jordan showing the highest contamination rate (17.6%) and Iraq the lowest (1.6%).^9^ Given the regional dietary reliance on dairy products, these findings underscore the need for enhanced food safety regulations and increased surveillance to prevent outbreaks.

### 4.7 Public Health Implications

From a public health perspective, our data support the need for more stringent food safety standards, well-directed educational initiatives, and regular food control activities in order to prevent listeriosis. Since pregnant women, the elderly, and immunocompromised individuals are most at risk, educational initiatives should be aimed at them, with an emphasis on the avoidance of raw milk, raw sprouts, and processed meats.^14–16^ In addition to avoiding unpasteurized dairy products and undercooked meat, public health campaigns should emphasize the importance of thoroughly cooking food, particularly animal-based products, to safe internal temperatures to reduce the risk of *L*. *monocytogenes* contamination.^22,23^ Thus, greater diagnostic sensitivity and earlier testing for at-risk populations may also help to lessen the consequences of this highly fatal infection.

### 4.8 Strengths and Limitations

This study represents the first comprehensive report on listeriosis in Qatar, offering region-specific insights over a decade. The use of laboratory-confirmed bloodstream infections enhances diagnostic reliability. Inclusion of a diverse expatriate population broadens applicability across Gulf states. However, the retrospective single-center design introduces risks of selection and information bias. Limited documentation of food history, lack of molecular typing, and the small sample size constrain generalizability and mechanistic insights.

## 5. CONCLUSION

This study provides critical insights into the epidemiology, clinical features, and outcomes of listeriosis in Qatar, revealing unique regional patterns alongside global trends. The findings highlight a higher proportion of pregnancy-related cases, rapid bacteremia clearance, and relatively lower CNS involvement compared to international data. While maternal survival rates were high, significant fetal loss underscores the need for targeted preventive strategies for pregnant women. Mortality was higher in older patients and those with CNS involvement, emphasizing the importance of early recognition and aggressive management of high-risk cases. The study also identifies gaps in antimicrobial resistance surveillance and foodborne transmission dynamics in the Middle East. To mitigate the impact of listeriosis in Qatar and the broader region, future research should focus on molecular characterization, source attribution, antimicrobial resistance monitoring, and long-term patient outcomes. Strengthening food safety policies and public health interventions will be crucial in addressing these challenges and improving infection control measures.

## LIST OF ABBREVIATIONS

**Table:** 

CLSI	Clinical and Laboratory Standards Institute
CKD	Chronic kidney disease
CNS	Central nervous system
CRP	C-Reactive Protein
HMC	Hamad Medical Corporation
ICU	Intensive care unit
IQR	Interquartile range
LOS	Length of stay
MALDI-TOF	Matrix-Assisted Laser Desorption Ionization-Time of Flight
MRC	Medical Research Center
PPI	Proton pump inhibitor
SPSS	Statistical Package for the Social Sciences
WBC	White blood cell

## DATA AVAILABILITY

The datasets generated and analyzed during this study are available upon reasonable request from the corresponding author, subject to institutional and ethical approvals.

## STATEMENT OF ETHICS

This study was conducted in accordance with the principles of the Declaration of Helsinki and was approved by the Institutional Review Board (IRB) under the Medical Research Center (MRC) of Hamad Medical Corporation (HMC), Qatar (MRC-01-21-1023).

## FUNDING INFORMATION

The research reported in this publication was supported by Medical Research Center (MRC) of Hamad Medical Corporation (HMC), Qatar under grants number MRC-01-21-1023. The research reported in this publication was supported by Qatar National Library under QNL open access program.

## ACKNOWLEDGEMENTS AND DISCLOSURES

The authors express their gratitude to the Medical Research Center (MRC) at Hamad Medical Corporation (HMC) for supporting this study. Special thanks to the microbiology and infectious diseases departments for their assistance in data collection and analysis.

## PATIENT CONSENT

A waiver of consent was obtained from the Medical Research Center (MRC) of Hamad Medical Corporation (HMC), Qatar, as the study involved only retrospective data collection without direct patient interaction.

## CONFLICTS OF INTEREST

The authors declare no conflicts of interest related to this study.

## Figures and Tables

**Table 1.** Demographic and clinical characteristics of patients with listeriosis in Qatar for the period May 1, 2011, to November 26, 2021 (*n* = 35).

Variables		Total (*N* = 35)
Female; gender		27 (77.14%)
Age (years; median, interquartile range)		39 (28–62)
World Health Organization’s region of origin	Eastern Mediterranean Region	23 (65.71%)
	South-East Asia Region	6 (17.14%)
	Western Pacific Region	1 (2.86%)
	European Region	1 (2.86%)
	African Region	4 (11.43%)
Hospital ward	Medical	19 (54.29%)
	Maternity	16 (45.71%)
Coexisting medical conditions	Diabetes mellitus	14 (40%)
	Chronic kidney disease	10 (28.57%)
	Cardiovascular diseases	6 (17.14%)
	Cerebrovascular diseases	2 (5.71%)
	Active malignancy	9 (25.71%)
	Chronic liver disease	6 (17.14%)
	Chronic lung disease	6 (17.14%)
Comorbidity and severity scores	Charlson Comorbidity Index (median, IQR)	2 (0–6)
	Pitt Bacteremia Score (median, IQR)	1 (0–2)
Risk factors	Age over 60 years	10 (28.57%)
	Gastrointestinal symptoms	4 (11.43%)
	Consumption of unpasteurized milk, cheese, or meat	1 (2.86%)
	Pregnancy	8 (22.86%)
	Malignancy	9 (25.71%)
	Dialysis	7 (20%)
	Use of proton pump inhibitors in the last 30 days	13 (37.14%)
	Organ transplantation	4 (11.43%)
	Other immunosuppressive conditions	9 (25.71%)

**Table 2.** Microbiological findings and clinical outcomes of patients with *Listeria monocytogenes* infection in Qatar for the period May 1, 2011, to November 26, 2021 (*n* = 35).

Variables			Total (*N* = 35)
Complications	Meningoencephalitis		7 (20%)
	Abortion (pregnancy-related cases)		2 (25%)
	Premature delivery (pregnancy-related cases)		5 (83%)
	No complications		21 (60%)
Length of hospital stay in days (median, IQR)			4 (2–7)
Bacteremia clearance within 72 hours			28 (80%)
Appropriate empirical antibiotic treatment			11 (31.43%)
Mortality rates		Mortality by day 30	1 (2.86%)
		Mortality by day 90	5 (14.26%)
Antimicrobial sensitivity (as per CLSI guidelines)^18^		Ampicillin	35/35 (100%)
		Co-trimoxazole	20/20 (100%)
		Meropenem	8/8 (100%)
		Gentamicin	1/7 (14.29%)

**Table 3.** Clinical characteristics and outcomes of pregnancy-associated listeriosis in Qatar for the period May 1, 2011, to November 26, 2021 (*n* = 8).

Case	Trimester	Singleton/twin	Initial appropriate antimicrobials	Positive placenta cultures	Definite antimicrobials /duration	Premature labor	Fetal outcome	Maternal outcome
1	3rd	Twin	No	Yes	Ampicillin/14 days	Yes	One survived (Term) One stillbirth	Recovered
2	3rd	Singleton	No	NA	Ampicillin + Gentamicin /11 days	Yes	Survived/preterm	Recovered
3	3rd	Singleton	No	NA	Ampicillin/14 days	Yes	Survived/preterm	Recovered
4	3rd	Singleton	No	NA	Ampicillin/14 days	No	Survived/preterm	Recovered
5	3rd	Singleton	Yes	NA	Ampicillin + Gentamicin/14 days	Yes	Survived/preterm	Recovered
6	2nd	Singleton	No	No	Ampicillin/10 days	-	Stillbirth	Recovered
7	2nd	Singleton	No	Yes	Ampicillin/10 days	-	Stillbirth	Recovered
8	3rd	Singleton	No	No	Ampicillin/10 days	Yes	Survived/preterm	Recovered

**Table 4.** Comparative analysis of survivors versus non-survivors after 1 year of *L. monocytogenes* infection in Qatar for the period May 1, 2011, to November 26, 2021 (*n* = 35).

Characteristics	Survivors (*n* = 30)	Non-survivors (*n* = 5)	*p* value
Median age (years)	36	63	0.003
Chronic kidney disease (CKD)	20.00%	80.00%	0.002
Meningoencephalitis	13.33%	60.00%	0.005
Intensive care unit admission	3.33%	80.00%	<0.001
